# Factors affecting farmers’ willingness and ability to adopt and retain vitamin A-rich varieties of orange-fleshed sweet potato in Mozambique

**DOI:** 10.1007/s12571-018-0845-9

**Published:** 2018-10-25

**Authors:** Mica Jenkins, Carmen Byker Shanks, Roland Brouwer, Bailey Houghtaling

**Affiliations:** 1 Food and Health Lab, Montana State University, Bozeman, MT USA; 2 International Potato Center, Maputo, Mozambique; 3 Department of Human Nutrition, Foods, and Exercise, Virginia Polytechnic Institute and State University, Blacksburg, VA USA

**Keywords:** Orange-fleshed sweet potato, Vitamin A deficiency, Nutrition, Farmers, Food-based approach, Food environment

## Abstract

The addition of orange-fleshed sweet potato (OFSP) to the food environment is an effective nutrition-sensitive agricultural approach to improve vitamin A intakes. However, the adoption of this biofortified crop merits further study. The objective of our research was to understand factors that affect Mozambican farmers’ adoption and retention of OFSP varieties, with a specific interest in the retention of planting material. Field research was conducted in three provinces of Mozambique during 2015. Provinces with different OFSP intervention histories were selected to allow for the identification of site-specific factors and the impact of variable approaches over time. Qualitative inquiry was used to assess participants’ progress through the five stages of the Innovation-Decision process in the Diffusion of Innovations Theory. Ninety-five producers, consumers, and market stakeholders of OFSP participated in semi-structured in-depth interviews and focus groups. Results indicate that diverse factors influenced the adoption and retention of OFSP, including organoleptic qualities, taste preferences, access to planting material, agronomic traits, environmental conditions, lack of capital for inputs and labor, unstable markets, and limited sharing of information and planting material across farmer networks. Current OFSP varieties were acceptable to Mozambican farmers and consumers, but there are several remaining challenges to reaching a critical mass such as lack of access to planting material, perceptions of superior drought tolerance of white-fleshed sweet potato (WFSP), and the belief that OFSP requires additional effort to cultivate (e.g. weed removal, measuring space between plants). Key recommendations which may be considered in future planning for OFSP interventions in Mozambique and other countries include enabling decentralized vine multipliers to provide vines to community members at no cost, continued focus on breeding and distribution of more drought tolerant varieties of OFSP, and training on the similarities in agronomic practices required for producing and preserving OFSP and WFSP.

## Introduction

Vitamin A deficiency (VAD) affects over 200 million women and children worldwide (WHO 2009) and 71% of children between six and 59 months of age in Mozambique, despite periodic distributions of vitamin A supplements (Aguayo et al. 2005). Lack of this essential micronutrient can lead to weakened immune systems, growth limitations, xerophthalmia leading to blindness, and increased mortality (Sommer and West 1996). The risk of death for children under age five with VAD is 1.75 times more than for children without VAD (Ross 1996).

VAD is a highly preventable condition that can be mitigated through the consumption of foods which contain preformed vitamin A, in the form of retinol, or beta-carotene, a precursor to vitamin A (Low et al. 2000; Bai et al. 2011). Retinol is derived from animal foods and beta-carotene is found in plant foods, including orange-fleshed sweet potato (OFSP). OFSP has been utilized as an important food-based intervention in geographic areas with chronic VAD (Hotz et al. 2012; Low et al. 2005, 2007a). Dark orange varieties of OFSP are higher in beta-carotene than many other commonly consumed foods, including mango, papaya, pumpkin and green leafy vegetables (Hotz et al. 2012).

Sweet potato (SP) has long been an important staple food for many Mozambicans, and thus the introduction of vitamin A-rich varieties as an amendment to the food environment has a relatively high chance of acceptability. The Portuguese introduced white-fleshed sweet potato (WFSP) to Africa in the sixteenth century (O’Brien 1972). Today, it is an important energy-dense food security crop in Mozambique (Minde and Jumbe 1997), where cyclical drought and flood are common (Kapinga et al. 2005). Economically, the sweet potato (SP) has been ranked as the third most important staple crop in Mozambique after cassava and maize (Walker et al. 2006); production levels for 2012 were estimated at 900,000 t (FAOSTAT 2015).

Generally, SP is a hardy crop that often succeeds when other staples fail (Woolfe 1992), making it especially important in areas prone to natural disasters. Sweet potato can also be produced multiple times in a year, with proper vine management and access to water. The crop is vegetatively-propagated, and during the dry season vine segments must be cut and replanted in moist lowland areas to prevent drying and loss of planting material. Sweet potato may also be regenerated from roots left in the fields during harvest which sprout when the annual rains begin. These preservation processes, when properly conducted over time, can lead to long-term retention of OFSP planting material in communities.

While the WFSP varieties commonly grown and consumed in Mozambique are low in beta-carotene (Low et al. 2000, 2007b; Mwanga et al. 2009), OFSP is rich in this precursor to vitamin A. Therefore, researchers and program designers determined that beta-carotene-rich varieties of OFSP have potential to fit within a nutrition-sensitive agriculture framework, where agricultural productivity and nutritional benefits can be simultaneously achieved (Herforth et al. 2012). Research has shown that an increase in intake of OFSP and other vitamin-A rich foods has a positive impact on serum retinol levels among women and children (Low et al. 2005, 2007a; Girard et al. 2017). Further, multiple authors have reported that OFSP is well-liked by Mozambican consumers and farmers (Low et al. 2005, 2007a; Hotz et al. 2012; Low et al. 2007b; Labarta 2009; Naico and Lusk 2010; de Carvalho et al. 2014), thus allowing the crop to be easily incorporated into current food environments.

Beta-carotene-rich varieties of OFSP were first introduced to Mozambique in 1997, when 38 varieties were received for testing at the Umbeluzi Research Station outside Maputo (Low et al. 2000). Since that time, a wide variety of government and non-government organizations, as well as international funders, have been involved in the breeding and promotion of OFSP in order to develop new varieties that are suitable for the various agricultural and cultural contexts found in Mozambique. Channels for promoting OFSP production, use, and consumption have included vine distributions, rural agricultural and nutrition extension workers, community training on improved agricultural practices and cooking methods, billboards, radio announcements, community drama, special market stalls for OFSP, and the distribution of bright orange *capulanas* (fabric), shirts, and hats (Low et al. 2007a, 2007b; Hotz et al. 2012).

Considerable progress has been made owing to fifteen years of research and collaboration by government agencies and the international public sector to promote OFSP as a food-based solution to VAD in Mozambique, including increases in vitamin A intake, decreases in VAD, and the 2011 release of 15 drought-tolerant varieties of OFSP bred in Mozambique. More information about the history and success of OFSP introduction and organizational involvement, including details on the Towards Sustainable Nutrition Initiative (TSNI) (Low et al. 2005, 2007a) and the scaled-up HarvestPlus Reaching End Users program (de Brauw et al. 2010; HarvestPlus 2012; Hotz et al. 2012; Jones and de Brauw 2015), can be found in Low et al. (2013), Low (2013), and Jenkins et al. (2015). However, there are several remaining challenges that have prevented this crop from reaching a ‘critical mass’, commonly accepted in social dynamics theory as the point at which an intervention becomes self-sustaining.^1^ These challenges include the preservation of planting material during the dry season and retention of OFSP during drought and flood, pest and disease management, market development for OFSP, and storage and processing for OFSP roots. The objective of our research was to use the Diffusion of Innovations Theory (Rogers 2003) to explore factors affecting Mozambican producers’ willingness and ability to procure, cultivate, preserve, and distribute OFSP varieties, with a particular interest in the retention of planting material seasonally and over time. The research team was also interested in the formal and informal interpersonal channels that may lead to the adoption or rejection of OFSP technology. The results of this research may be used to inform the design of future food-based approaches to alleviating micronutrient deficiencies in similar country contexts.

## Methods

### Research design

The variable adoption levels of OFSP in Mozambique directed the research team to apply the Diffusion of Innovations Theory (Rogers 2003) to determine the factors affecting the spread of OFSP and related technology throughout communities. This theoretical framework was used to guide the development of primarily qualitative methods, including semi-structured interviews and focus groups, coding, data analysis and interpretation in order to assess participant progress through the Innovation-Decision process.

Rogers (2003) analyzed adoption of innovation through five stages: knowledge, persuasion, decision, implementation, and confirmation (see Table 1). These five stages are referred to collectively as the Innovation-Decision process and include exposure to the technology or idea, opinion formation, experimentation with and use of the new technology or idea, and finally the decision whether or not to continue using the technology or idea in the long-term. Furthermore, relative advantage, similarity with current practices, ease of use, ability to experiment before adoption (i.e., trialability), and tangible results of an innovation influence the pace of adoption and the decision-making processes of current and potential producers, consumers, and market stakeholders (Rogers 2003). For example, because WFSP varieties are widely grown in Mozambique (Low et al. 2000, 2007b), OFSP varieties are not an entirely new technology, and are believed to be at an advantage for adoption due to similarity with current practices.

**Table 1:** Measures for assessing participant progress through five stages of the Innovation-Decision process to understand factors affecting farmers’ willingness and ability to adopt and retain vitamin A-rich varieties of orange-fleshed sweet potato in Mozambique

Diffusion of innovations stage	Measure
Knowledge: exposure to an innovation and understanding of how it functions	When and how did you learn about OFSP? When did OFSP first appear in markets?
Persuasion: formation of favorable or unfavorable attitude toward the innovation	Why is SP (OFSP and/or WFSP) important in your community? Is SP a respectable crop? How are WFSP and OFSP similar and/or different?
Decision: engaging in activities that lead to a choice to adopt or reject the innovation	When and how did you begin to produce OFSP? Why did you start? When did you first eat OFSP?
Implementation: putting an innovation to use	What is the area of your OFSP production? Are you a landowner? How is OFSP used in your home? Are SP leaves used in your home? Which OFSP varieties are used in your community? Who decides how OFSP will be produced, harvested, and sold? (Man, woman, child?) How do you plant OFSP and WFSP? Together or separately? Do you use fertilizers, tractors, and/or hired laborers? How many times a year do you plant and harvest WFSP and OFSP? How are vines preserved? What is the post-harvest process for roots? Do you have storage for SP? Is there a difference in the amount of water needed for OFSP and WFSP? Which is more resistant to sun? To insects? Which SP produces faster? Is there a difference in the work involved with OFSP and WFSP? Is one SP more difficult to produce than the other? Why does planting material sometimes disappear?
Confirmation: seeking reinforcement of decision already made; may reverse previous decision if exposed to conflicting messages about the innovation	What is the SP that you and your family prefer? Have you shared OFSP with other people? How can a person obtain OFSP vines? Do you sell SP? Where? What is the price of OFSP and WFSP? Which is the SP that market clients prefer? How can SP projects improve in the future?

### Setting

Field research took place from January to October 2015 in three districts of three provinces in Mozambique that differ in terms of agro-ecology, culture, socioeconomic realities, market norms, and intervention history: Manhiça in Maputo Province, Macate in Manica Province, and Gúruè in Zambézia Province (see Table 2). The sites were chosen on the basis of the following parameters: (1) the occurrence of recent OFSP promotion efforts, such as International Potato Center (CIP) vine distribution activities through the Scaling Up Sweet Potato Through Agriculture and Nutrition (SUSTAIN) project, and/or; (2) previous distributions of OFSP planting material conducted by CIP and other organizations including ActionAid, Africare, and World Vision. Data were collected for one month in each of the three research sites through in-depth semi-structured interviews and focus groups (Morgan 1997).

**Table 2:** Characteristics of research sites in Mozambique

District, province	Geographic context	Population	Key commodities	OFSP interventions past and present^e^
Manhiça, Maputo	80 km northeast of Maputo city	160,539^a^	maize, cassava, groundnut^b^	CIP-SUSTAIN^f^ (current); ActionAid (2001 forward)
Macate, Manica	25 km east of Chimoio city	Newly designated district, population data unavailable	maize, sorghum, cassava^c^	CIP-SUSTAIN (current); CIP-OFDA^g^ disaster relief effort (2012–2013); Africare (2002–2006)
Gurúè, Zambézia	In the Namuli Mountain Range	299,565^a^	cassava, maize, sweet potato^d^	REU^h^ (2006–2009); Eat Orange (2006)

^a^*Recenseamento Geral da População e Habitação*. Maputo, Moçambique: Instituto Nacional de Estatística; 2007

^b^MAE. (2005a). Perfil do distrito de Manhiça. Maputo, Ministério de Administração Estatal and Métier

^c^MAE. (2005b). Perfil do distrito de Gondola. Maputo, Ministério de Administração Estatal and Métier

^d^MAE. (2005c). Perfil do distrito de Gúruè. Maputo, Ministério de Administração Estatal and Métier

^e^Not an exclusive list. For more information about OFSP interventions see Low et al. 2013, Low 2013, Jenkins et al. 2015

^f^International Potato Center project Scaling-Up Sweetpotato Through Agriculture and Nutrition

^g^A collaborative effort by the International Potato Center and the Office of Foreign Disaster Assistance of the United States Agency for International Development

^h^Reaching End-Users project in Zambézia

Manhiça has a dry subtropical climate with an annual rainfall of approximately 800 mm and one rainy season concentrated between December and February. About 60% of the landholdings are smaller than 1 ha and the average size is 1.2 ha. The main staples are maize, cassava, and groundnut. The district is located close to the coast on the Incomáti flood plain, which is dominated by alluvial, clay soils; most SP farming takes place in the flood plain. Seasonal flooding as well as long dry spells are the main climatic factors affecting SP farming (MAE 2005a); vines may be conserved in the more humid areas of the river valley.

Macate has an elevation of approximately 500 m above sea level with a moderately humid tropical climate. The district receives approximately 1000–1500 mm of rainfall per year, principally during the rainy season from November to March. Its soils are deep, red, sandy to clay soils (laterite). Of the landholdings in the district, 44% are less than 1 ha, and approximately 39,000 have an average size of 1.5 ha; the main staple is maize (MAE 2005b). The landscape is undulating and households may have access to low-lying land next to rivers and catchments where SP vines can be conserved.

The elevation in Gúruè varies between 500 and 1000 m above sea level. The district has a tropical humid climate modified by the inselbergs that dominate the landscape. Annual rainfall in the district is approximately 2000 mm (though significantly lower in some areas) and is concentrated between November and April. Soil types vary; red laterites are the most common. Approximately 72% of the holdings are smaller than 1 ha. The main staples are cassava, maize and SP; tea is also an important cash crop for the district (MAE 2005c).

### Sample

The research team employed three purposive sampling strategies to recruit interview and focus group participants: critical case sampling of participants with specific experiences; key informant sampling of participants with special expertise; and snowball sampling of participants identified by other informants (Marshall 1996). Participants were identified by: contacting individuals who had been involved with CIP, including current staff, decentralized vine multipliers (DVMs), and project beneficiaries; seeking the assistance of local government extension workers; engaging in conversations with the leaders of farmers’ associations; visiting local markets, and; through word-of-mouth.

### Qualitative methods

Basic demographic information was gathered through a simple questionnaire (see Table 3). The script for in-depth semi-structured interviews and focus groups was designed to assess individual progress through the five stages of the Innovation-Decision process (see Table 1).

**Table 3:** Demographic characteristics of participants in interviews and focus groups

Variable	Mean (±SD) or %
Region	Manhiça = 26.3% Macate = 50.5% Gurúè = 23.2%
Age	Sample = 39.1 (±15.2) Female (F) = 35.1 (±11.8) Male (M) = 45.7 (±17.8)
Sex	F = 63.2% M = 36.8%
Married^a,b^	Sample = 81.1% F = 76.7% M = 88.6%
Number of children	Sample = 4.7 (±2.9) F = 4.2 (±2.5) M = 5.5 (±3.5)
Education level (number of years)	Sample = 5.0 (±3.1) F = 4.2 (±3.1) M = 6.4 (±2.7)
Occupation	Producer = 87.4% Vendor = 7.4% Other = 5.3%
Holds a leadership position^c^	Sample = 42.1% F = 25% M = 71.4%
Involved in community organizations	Sample = 93.7% F = 95% M = 94.1%

^a^The remainder of the sample was unmarried (9.5%) or widowed (9.5%)

^b^Married includes those who identified themselves as “Married.” Unmarried includes those that answered the question “Are you married?” with a negative response

^c^Participants who responded positively to the question “Are you a leader in the community?” were counted in this sample

Interviews and focus groups were conducted at or nearby participant homes or the homes of community leaders. When possible, focus group participants were segregated by gender to ensure that women could speak freely. When the researcher felt it would have been inappropriate to exclude those who demonstrated a desire to participate, or when there was not sufficient time or number of participants to segregate genders, the focus groups were conducted with both men and women (FG = 9).

Interviews and focus groups were conducted in Portuguese by the first author or through a translator in the local language, depending upon the context and the preference of the participants. Female participants used local language more often than male participants, which may be attributed to the fact that male participants averaged a greater number of years of formal education (see Table 3). All interviews and focus groups were recorded with participant verbal consent to ensure that all information was captured. The first author also kept a detailed field log with notes from interactions with NGO staff and government extension workers, informal conversations with OFSP stakeholders, market observations, plans for interviews and focus groups, and lists of recurring themes of research. Notes from this log have been triangulated with findings from interviews and focus groups, contributing to the overall validity of results (Creswell 2014).

Qualitative data analysis was ongoing as interviews and focus groups proceeded. Interviews were de-identified for anonymity, transcribed verbatim, and translated into English by the first author. The first author chose to translate interviews from Portuguese into English simultaneously while transcribing due to fluency with the Portuguese language and also to ensure efficient use of limited time and resources (Temple and Young 2004). Meaning units (MUs) (distinct data constituting a single idea), were separated into fragments, organized in a coding template developed by the researchers (Krippendorff 2004), and analyzed at the fragment level, as opposed to the participant level, to ensure a common understanding of the emerging findings. Once all interviews were complete, authors independently coded the fragments for appropriate themes and subthemes, using a deductive approach and the five stages of the Innovation-Decision process as main constructs (Rogers 2003). Coding authors worked from a codebook of 79 subthemes developed by the first author and agreed upon by co-authors, while also allowing for additional subthemes to emerge during coding. Once independent coding was complete, the first author took note of all discrepancies between coders in the constructs (*N* = 1105) and subthemes (*N* = 1088), suggesting a resolved construct and subtheme for each MU. These resolved categories were then approved or rejected by the other coding authors, resulting in a smaller number of discrepancies in the constructs (*N* = 0) and subthemes (*N* = 13) that were resolved by coding authors. Finally, the 79 original subthemes were discussed by co-authors and collapsed into 23 themes, detailed in the results section and figures. This rigorous coding process ensured a high-level of inter-rater reliability (Wertz 1983). Data were disaggregated by research site and the number of MUs that appeared in each category and subcategory was calculated. Findings from the three locations are compared below to provide insight into how varying factors in these locations may affect the adoption or rejection of OFSP technology.

## Results

**Fig. 1:**
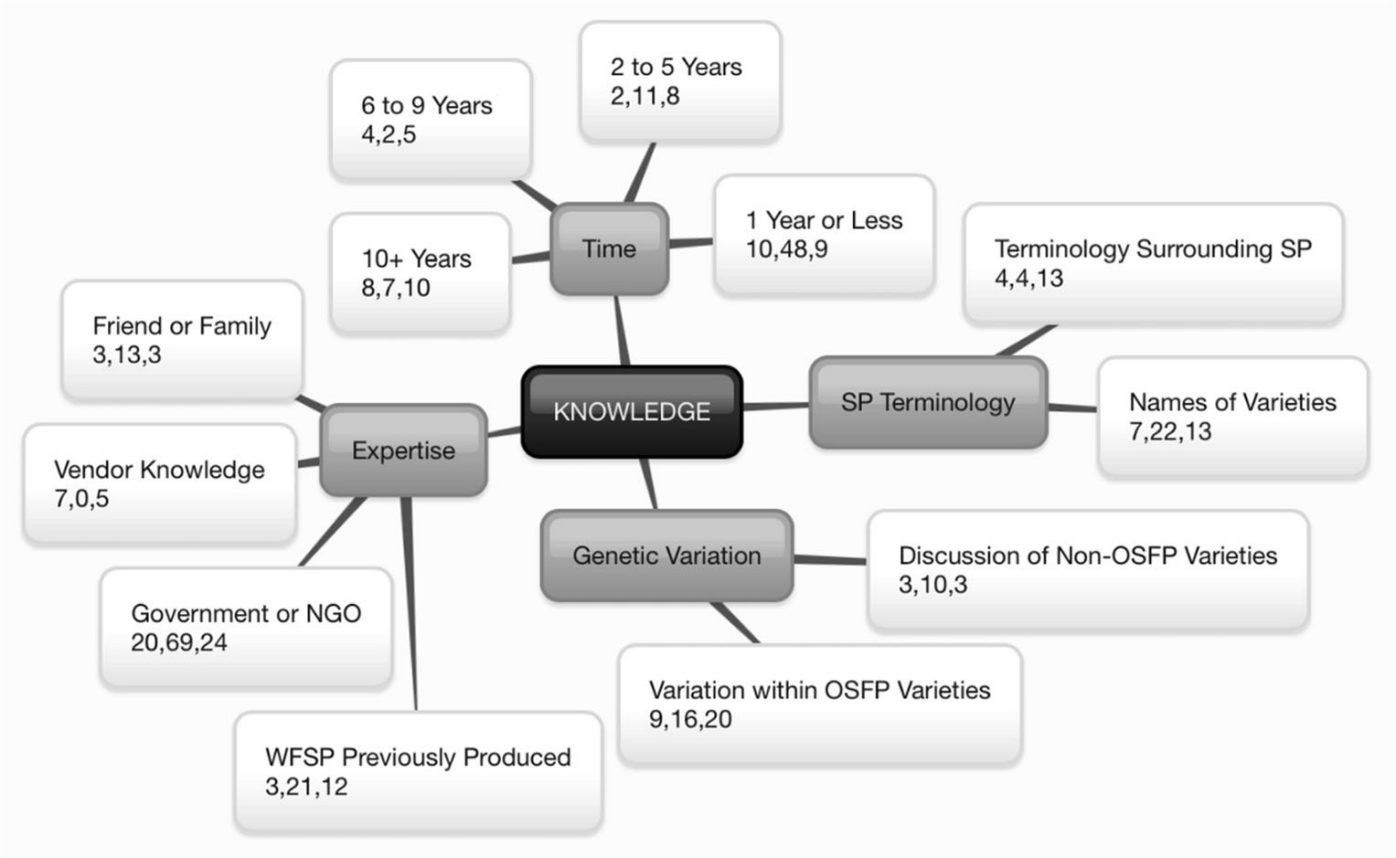
Knowledge^+^ of Orange-Fleshed Sweet Potato Adoption and Retention in Mozambique^*. +^Knowledge characterized through Diffusion of Innovations (Rogers 2003): exposure to an innovation and understanding of how it functions. ^*^The center box represents the construct. First-level branches represent identified themes. Second-level branches represent identified subthemes. Within the second-level branches, numbers represent the number of quotes collected from participants in: (1) Manhiça in Maputo Province, (2) Macate in Manica Province, and (3) Gúruè in Zambézia Province

**Fig. 2:**
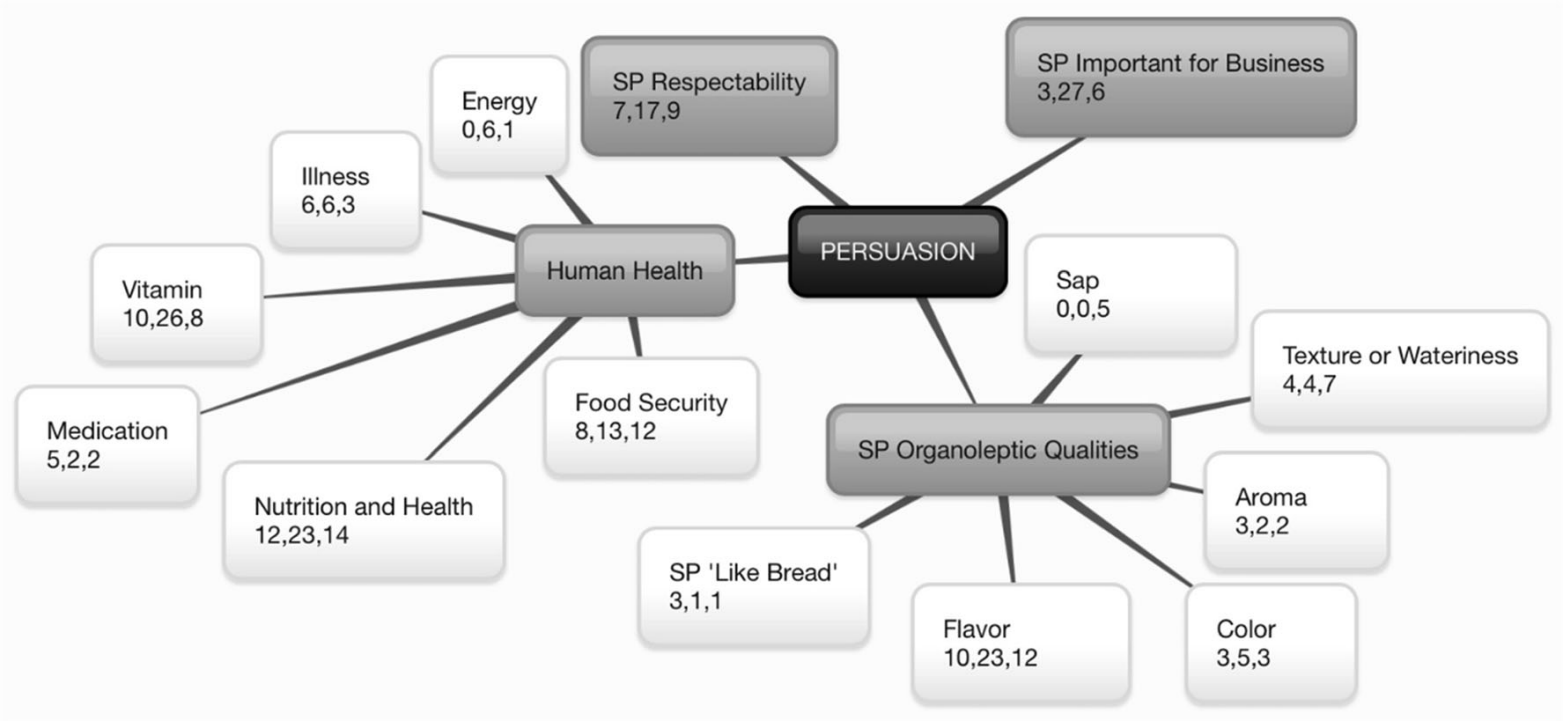
Persuasion^+^ of Orange-Fleshed Sweet Potato Adoption and Retention in Mozambique^*. +^Persuasion characterized through Diffusion of Innovations (Rogers 2003): formation of favorable or unfavorable attitude toward the innovation.^*^The center box represents the construct. First-level branches represent identified themes. Second-level branches represent identified subthemes. Within the first and second-level branches, numbers represent the number of quotes collected from participants in: (1) Manhiça in Maputo Province, (2) Macate in Manica Province, and (3) Gúruè in Zambézia Province

**Fig. 3:**
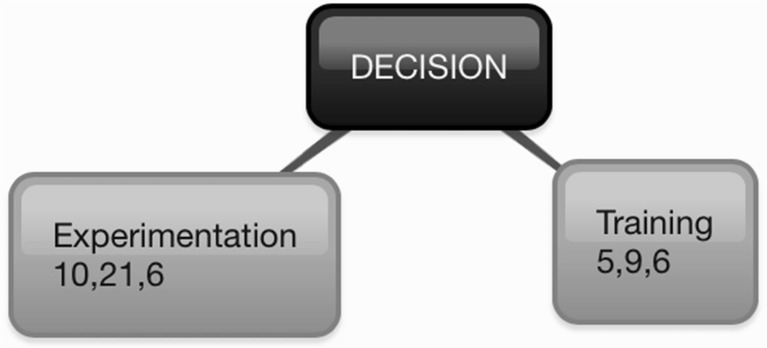
Decision^+^ of Orange-Fleshed Sweet Potato Adoption and Retention in Mozambique^*. +^Decision characterized through Diffusion of Innovations (Rogers 2003): engaging in activities that lead to a choice to adopt or reject the innovation. ^*^The center box represents the construct. First-level branches represent identified themes. Within the first-level branches, numbers represent the number of quotes collected from participants in: (1) Manhiça in Maputo Province, (2) Macate in Manica Province, and (3) Gúruè in Zambézia Province

**Fig. 4:**
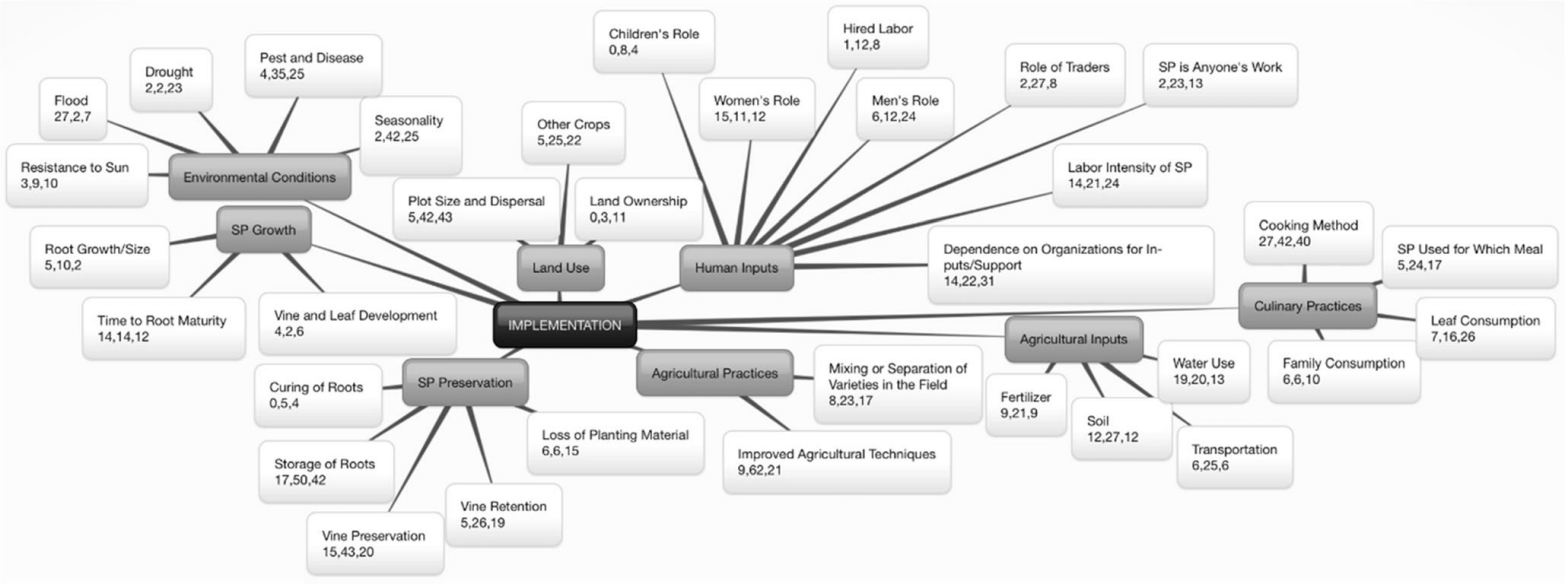
Implementation^+^ of Orange-Fleshed Sweet Potato Adoption and Retention in Mozambique^*. +^Implementation characterized through Diffusion of Innovations (Rogers 2003): putting an innovation to use. ^*^The center box represents the construct. First-level branches represent identified themes. Second-level branches represent identified subthemes. Within the second-level branches, numbers represent the number of quotes collected from participants in: (1) Manhiça in Maputo Province, (2) Macate in Manica Province, and (3) Gúruè in Zambézia Province

**Fig. 5:**
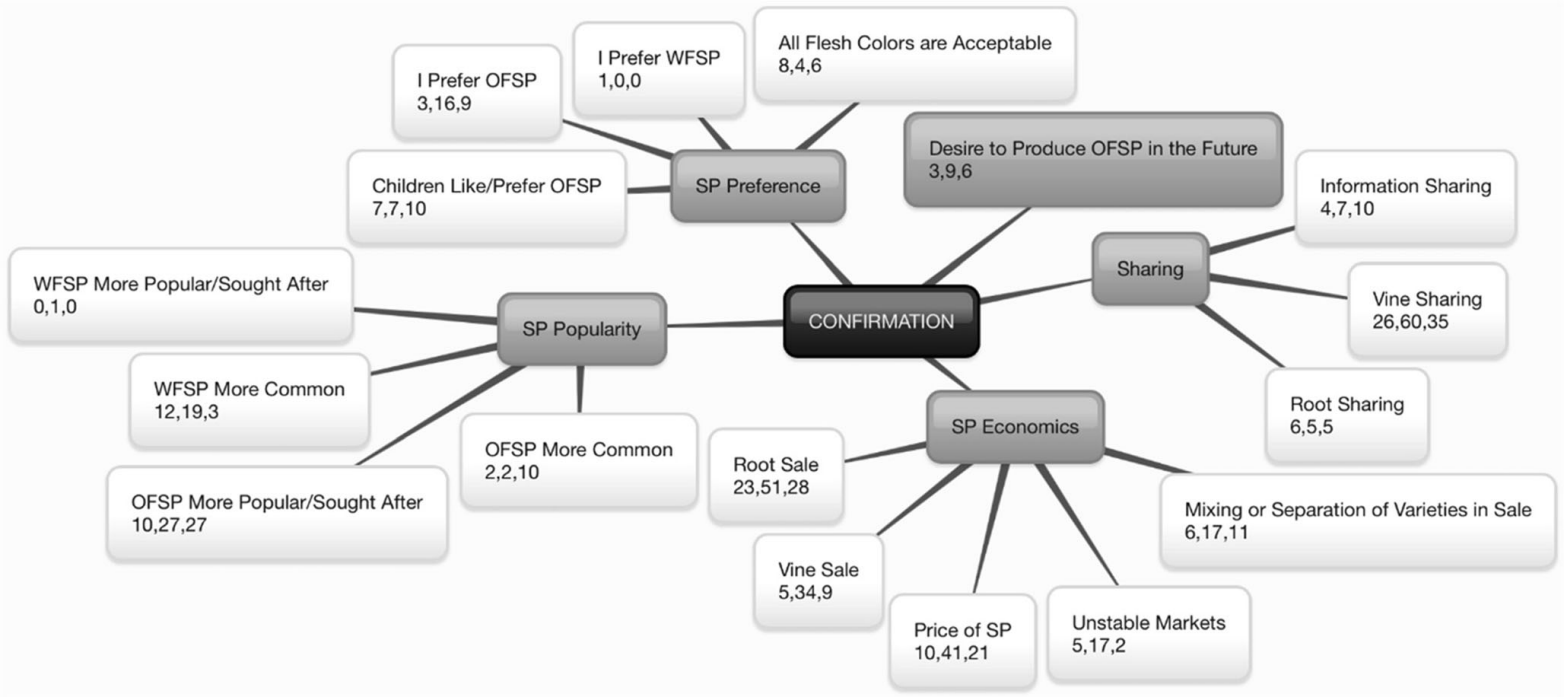
Confirmation^+^ of Orange-Fleshed Sweet Potato Adoption and Retention in Mozambique^*^. ^+^Confirmation characterized through Diffusion of Innovations (Rogers 2003): seeking reinforcement of decision already made; may reverse previous decision if exposed to conflicting messages about the innovation. ^*^The center box represents the construct. First-level branches represent identified themes. Second-level branches represent identified subthemes. Within the second-level branches, numbers represent the number of quotes collected from participants in: (1) Manhiça in Maputo Province, (2) Macate in Manica Province, and (3) Gúruè in Zambézia Province

Participants included OFSP producers, vine multipliers, consumers, and market stakeholders with varying levels of exposure to and experience with OFSP. Ninety-five participants (F = 60, M = 35) were included as individual informants (*N* = 15) or focus group participants (*N* = 80, FG = 15). Meaning units contributed by males and females were equally represented, regardless of imbalance in the number of participants. Interviews and focus groups ranged from 28 to 87 min and lasted an average of 55 min (SD ± 15.8). In total, 3015 meaning units emerged across 23 themes and 79 subthemes, all organized under the five Diffusion of Innovations constructs. Figures 1, 2, 3, 4, and 5 exhibit the final themes and subthemes categorized under each construct. Additionally, the number of meaning units generated from Manhiça, Macate, and Gurúè are shown in order to explore regional differences. Sample meaning units for selected subthemes are in Table 4.

**Table 4:** Sample meaning units organized by subtheme

Subtheme	Meaning unit (P#: refers to the participant number as coded for anonymity during the research)
Vitamin	P39: We learned that there is a difference…between white pulp and of orange pulp. Of orange pulp, it’s a potato that has vitamin, vitamin A? Translator for P70: ...because we heard that the same potato…the vitamin A, also, it makes children grow well with health… Then, also, it also gives…vision…
Medication	P75: In the actual potato, it serves also as medicine in your body, it works a lot.
Nutrition and health	P1: I have to eat this potato. Because I know in the body it’s good for me. P36: It makes good health for people. P39: It’s good also for the children.
Food security	P83: The orange pulp, at times, during two, two months, it already has tubers below. It can come to help in the time, already that we’re going in the time of hunger…While this…from here, regional…It takes longer. It can take around six months.
Sweet potato ‘like bread’	P82: Yes, for this, it’s our bread here. Translator for P4: He says, as they don’t have a bakery here, eh, close, it’s the quicker bread, accessible, that you can find.
Flavor	P50: Eh, the difference maybe is in the flavor. That one seems very…a little sweet in relation to…our white one. Translator for P56: She says that orange pulp is better…It’s better in the flavor, it has flavor. Translator for P60: This of orange pulp, the flavor is very good…
Sap	P75: Because, another thing that the people noted, they saw that that white pulp had a thing that seemed like glue, in the mouth. After you, eh, you eat it, it seemed already, it glued the lips…While this new varieties, no. P83: This local, when you eat it…here in the mouth, it has…sap here. Yes, who sees it is going to know that that person already ate sweet potato. While this orange pulp doesn’t bring anything here in the mouth.
Leaf consumption	P88: Because the white potato, when a person cooks it, the leaves don’t taste good. P89: Also, it has a thing that gets a little bitter. P88: Yes yes, now that there, that one that’s there, that leaf. It’s very tasty. That one there, orange pulp potato.
Soil	P29: We have a soil mixed with a bit of sand a bit of, of clay…it enjoys a lot more in that part, that soil that has…a bit of, of sand, a bit of clay. P93: It’s necessary a, a mixed soil. Yes. That red a bit also black. A mixture yes.
Water use	P13: Because they think it’s not going to produce well because there is a lack of water…While that other one [WFSP] doesn’t need water. P26: When the rain falls well. it’s orange flesh…it’s white, it just grows the same, it’s going to depend on the rain.
Women’s role	P66: A lot a lot, they are, they are the mothers. We are the mothers yes. Yes…Eh, these fathers say ‘eh we have a lot of work’, or ‘it’s not our work’.
Men’s role	P27: Men save seeds. Children, women, ah they don’t manage…Just, just good to eat only. To save, it’s men that save this crop, seeds.
Sweet potato is anyone’s crop	P74: They have to do, doesn’t matter if it’s man, doesn’t matter if it’s woman. If it’s woman, if it’s not woman, you have to do it if you have strength in your body.
Labor intensity of sweet potato	Translator for P35 and P36: It’s to say that white pulp is easy in relation to orange pulp, because the orange pulp needs…maintenance…attention there. Translator for P37: The white pulp, even with weeds, mixing the soil and weeds, it’s possible to put the, the vine. But as to the orange pulp, it’s necessary to prepare, take it out, take out those trunks, also roots of…weeds, it’s necessary to take it out, to have only clean soil.
Drought	P30: The drought, white resists more…For example here where you are seeing, I already planted, already four or five years passed, when I did potato here. But I try to weed for it to come out, it doesn’t come out. No no…Yes it resists…Here I haven’t planted, already five years have passed...Five years vines, but I am accustomed to try, ‘epa’, to weed and maybe it already passed, but it’s going to germinate more…It’s strong, it resists more.
Flood	Translator for P21: Then his problem, of our zone, is that, we are producing, but, what makes us sad is that, when you produce, annually, floods have to come. Take all our crops.
Resistance to sun	Translator for P55: If you harvest and don’t take out the [OFSP] vines to go put in the lowlands, where there is moisture, it doesn’t manage to endure outside. While the white pulp endures outside.
Root growth/size	P50: That orange pulp, because at times it develops…it is longer, no? The actual potato is longer…While this white pulp…it’s more round. P49: [WFSP] it’s just voluminous…But that orange pulp, eh, it’s long.
Time to root maturity	P30: The difference that exists, this white pulp, when we plant it, it delays. Mm? It delays to arrive to the time to take it out. While orange pulp no. When you plant it, after a month, it’s already two, when you take it out like this…it already has potato below…this white no. It has to stay there the same.
Vine and leaf development	P74: Orange pulp doesn’t need a lot of water…Now this white pulp, it doesn’t manage, because it takes a lot of vines…They are longer, they go farther, and they are big…They’re thick and they have many vines…They need water…They [OFSP vines] are smaller.
Improved agricultural techniques	P13: The care, if you produce orange sweet potato, in any form, doesn’t produce that fruit that you needed. You need to control…apply four vines. In a hole. It’s what we grew up doing. But while this one here, it’s just with one vine. Afterward you take out 4 or 5 big potatoes. You see what is the difference. P47: This orange pulp you measure…white pulp, it’s just to put it…you plant it, you don’t measure. P39: And to plant [OFSP], you have to measure, plant in respect, in his rules…white pulp, it’s just plant it in any way, we can’t measure anything. Planting in any way it also comes out, it doesn’t…have a lot of complication.
Mixing or separation of varieties in field	Translator for P15: They plant them together here, just that you shouldn’t put it in the same place. You have to separate. Because it’s not born in the same way…potato doesn’t come out at the same time. That one [OFSP] is fast, the other [WFSP] takes time.
I prefer OFSP	P74: The more tasty potato is this, orange pulp…it’s more tasty, flavorful. It brings more strengths… Than the white.
Children like/prefer OFSP	Translator for P77: When the children see that orange pulp potato, the child doesn’t leave anything, they eat it until it runs out. They eat it willingly, yes.
OFSP more popular/ sought after	Translator for FG2: It’s not easy, because, each person wants that pulp, because the pulp is good. It’s not like that, those [WFSP]. It’s not easy to find seeds also. P2: And when we sell OFSP, and that one ours…what goes out as fast as possible is the OFSP, that goes out as fast as possible. Than that our one.
Price of sweet potato	P31: For example if it’s that first time there, first time…November…then, you can make at least 500, 450 [meticais]. The price, one sack like that there…Then, this group of red [skin], you can sell 300, 250 [meticais]…But orange pulp doesn’t accept even to be until 250…It has a lot of competition even… Always, we can say that…orange pulp always a price a little advanced.
Mixing or separation of varieties in sale	P30: Last year, when I produced a lot of potato, then, I went to mix. I mixed, it was, this white, orange pulp, then I mixed…Yes inside the sack. When I arrive in [market] 38, they grabbed that sack, potato, potato, after, they went to see, then dumping it out…‘Eh, Mama, only potato no no, we’re going to choose…I only want orange pulp…Then I went to see that ah, it doesn’t have advantage this white…Now I already don’t mix. P89: Yes, you mix. This and this, no problem.
Vine sharing	P26: Ah, here in the community, it’s to give…It’s give because you, also in another year, you can lose, also you’re going to ask also from him…We work like this. P89: If you don’t have the vines, the time arrives, you can make your seed beds, go ask from any person, they give. P28: Ya, white, who wants to come take can just take even. It doesn’t have interest.

### Knowledge

Participants generally reported learning about OFSP through government extension workers or an NGO (MU = 113), while fewer reported learning through a friend or family member (MU = 19). In Macate, the majority of participants had received vines for the first time in 2014 or 2015 (MU = 48). In Gurúè and Manhiça, more participants reported having worked with OFSP for six or more years compared to Macate. There was a notable difference in interviewee awareness of OFSP and confidence in making SP derivatives in Gurúè compared to the other two districts, attributable to the intensive efforts of World Vision, HarvestPlus and partners to promote OFSP in Zambézia through multiple projects from 2003 to 2009 (Low et al. 2005; de Brauw et al. 2010).

Participants spoke at length of the variation between the OFSP varieties they have received (MU = 45), commenting on flavor (MU = 45), color (MU = 11), root size (MU = 17), and vine/leaf development (MU = 12). Such observations were especially common among DVMs, who often received six or more varieties for multiplication and community distribution. Some participants preferred specific OFSP varieties for specific preparation methods, such as juice and bread. Other participants shared information about specific yellow and white varieties (MU = 16). For example, in Macate, many farmers emphasized the importance of the WFSP variety known as *Secai*, which is considered to be drought tolerant, flavorful, and high in dry matter content.

The terminology used to describe both white and orange varieties of SP emerged as an important theme (MU = 21). White-fleshed sweet potato was also referred to as “our potato”, “the old potato”, “natural”, “local”, “regional”, “original”, or “traditional” while OFSP was referred to as a “novelty”, “new potato”, or “thing of honor”, indicating a lack of perceived ownership of OFSP.

Across sites, few participants, aside from DVMs, were able to correctly recall the names of the varieties of OFSP that they had received and were currently producing. Participants in Macate spoke somewhat more comfortably about variety names (MU = 22) than those in the other two districts, likely the result of the relatively recent introduction of OFSP in Macate. Participants in Gurúè reported that they had received the varieties *Irene* and *Gloria* during World Vision’s 2003 distributions, which was not possible as these varieties were released in 2011. Some interviewees reported that they renamed the varieties used in their communities rather than using the original names.

The terminology used to describe SP was regionally variable and often related to color. In Manhiça and Macate, orange varieties were referred to as “carrot potato”, while participants in Gurúè were unfamiliar with this colloquialism. One participant in Macate also compared orange varieties to pumpkin. Yellow or orange potatoes were also sometimes referred to as “egg potato” in Portuguese and in local language, due to the similarity in color with egg yolk. In Gurúè, the local names for “orange sweet potato” translated to “egg potato” or “orange” or “red potato”. The spectrum of what was considered by some to be a “yellow” potato was broad, ranging from very light yellow to medium orange. While some simply used the word “yellow” to describe any flesh color that was not white, those who had been exposed to the terminology “orange-fleshed sweet potato” often distinguished between orange and yellow.

### Persuasion

Participants in all districts were familiar with OFSP and its relative advantage over WFSP in terms of nutritional value (MU = 49) and vitamin content (MU = 44); healthy eyes and vision were reported less frequently and, therefore, did not emerge as a subtheme. Participants reported that OFSP was important for food security (MU = 33) and could also be used to prevent or treat illness (MU = 15). Several participants and market informants compared OFSP to medication (MU = 9), a belief that stems from the fact that it is promoted as a food to help prevent blindness and to promote immune health as well as proper growth and development, especially in pregnant women and young children.

Several interviewees compared SP to bread (MU = 5), indicating that it was an important substitute in communities that do not have access to a bakery or the ingredients to make bread. It is often consumed as an accompaniment to tea, as bread would be, and is considered a snack food that is quick and easy to prepare. Several participants noted a pleasant aroma associated with cooking OFSP roots (MU = 7). Participants in Macate and Gurúè noted negative side effects of eating WFSP leaves and roots, including a bitter taste, stomach pain, and sores on the lips or “gluing” of the lips resulting from contact with SP sap, due to the presence of latex. These participants reported that consuming OFSP does not provoke such discomforts, further contributing to perceptions of relative advantage.

In all districts, and particularly in Macate (MU = 27), participants emphasized that SP was increasingly seen as a crop for business and not just home consumption, especially OFSP which was reported to be of higher value and more competitive than WFSP. Participants in this study stated that SP was respectable and they enjoyed producing and sharing it, contrasting previous research that suggested SP may be perceived as a poor person’s crop (Brito et al. 2012).

### Decision

Participants reported that they had received training from the government or an NGO on how to produce and prepare OFSP for consumption (MU = 20); engaging in such activities is an important step in the decision-making process and may lead to implementation and eventual confirmation that the innovation is indeed useful.

It is clear that experimentation is a critical step in a farmers’ decision to adopt or reject OFSP (MU = 37). Several participants who were producing OFSP for the first or second time mentioned that they wanted to compare it to WFSP, especially in terms of drought tolerance and time to root maturity; others were keenly interested in obtaining more varieties of OFSP for experimentation and comparison. Participants reflected positively on cooking sessions organized by government or NGO workers that allowed them to compare the flavor and texture of a variety of OFSP roots and leaves.

### Implementation

Overall, cooking method was one of the most frequent subthemes of this research (MU = 109). Participants also frequently discussed the importance of SP leaves as part of the family diet (MU = 49). In an effort to increase OFSP intakes, CIP has followed the strategy to present SP as an alternative to ‘Irish potato’ to encourage consumption at lunch or dinner in addition to breakfast. This strategy may not be entirely compatible with Mozambican dietary norms as SP are generally consumed as a breakfast or snack food, which was confirmed by participants; some felt that since SP is a sweet food, it does not pair well with savory items such as fish or meat. However, a portion of participants reported that OFSP could be eaten at any time of day, and mentioned recipes which involved onion and tomatoes cooked with SP roots or leaves. Still others reported that SP might be eaten for lunch or dinner, but only when other more typical food items (i.e., maize, cassava) are not available.

Soil type (MU = 51) and water use (MU = 52) for the successful production of SP emerged as important subthemes. Multiple participants voiced concerns about SP production that also characterized their experience as subsistence farmers, in particular a lack of inputs to expand production, especially fertilizers (MU = 39), as well as transportation required to participate in the value chain (MU = 37). Some stated that they managed a successful SP crop without fertilizers, and several participants in Manhiça reported that the use of fertilizers on the crop negatively affected the flavor and texture of any variety of SP.

Reports regarding the responsibilities of men, women, and children in the production of SP varied substantially. Participants in all three districts reported that SP can be cultivated by both men and women (MU = 38); however, in each district, there were also a considerable portion of participants who believe that SP is a crop with gender-specific roles and responsibilities (see Fig. 4 for MUs). Two male participants in Macate, where SP is an important cash crop, noted that high levels of unemployment have led men of all ages to focus on SP for their livelihoods. Other men in Macate, and both men and women in Gurúè, reported that while anyone could grow SP, vine preservation was the responsibility of men. Other participants in Gurúè reported that men make the decisions regarding SP production and sale and are also largely responsible for farm preparation and vine preservation, while women mainly participate in the harvest. Still others in Macate and Gurúè reported that the sale of SP was a male responsibility, or that the woman becomes responsible only when the man has other work to do. However, women also reported high levels of responsibility for production, sale, and vine preservation, indicating themselves as autonomous decision-makers for this crop. Various women in Gurúè reported that while their husbands might be responsible for the production and vine preservation of SP, the women are responsible for the sale.

Participants in all districts, in particular Macate, discussed their experiences with traders (MU = 37). Some farmers were highly concerned that traders use deceptive techniques to drive down farm prices, including misrepresenting the supply and demand of SP at the nearest markets. Others reported that selling on-farm saves time but is less profitable. Farmers who attempt to transport their own SP to market for sale may also be intercepted in transit by traders who wish to purchase SP directly.

Participants discussed the labor intensity of all SP varieties (MU = 59) due to the need to make soil ridges, which limits the quantity they are able to plant, although this technique increased the yield per plant per square meter. Those who can afford to pay for hired labor may do so to increase the amount of SP they are able to cultivate (MU = 21). Others noted that the work of multiplying vines is difficult when beginning with a small amount of vines. While participants in all districts reported some level of dependence on NGOs and government for OFSP support, this subtheme was most pronounced in Gurúè (MU = 31), potentially due to the influence of the scaled-up HarvestPlus Reaching End Users project which was implemented in Zambézia from 2006 to 2009 (Hotz et al. 2012; de Brauw et al. 2010).

Interviewees in each region reported on the impacts of annual flooding (MU = 36), drought (MU = 27), and pests and disease (MU = 64) on SP and other crops. The negative impact of flooding was reported more often in Manhiça than in the other two districts (MU = 27). In Manhiça, OFSP is grown in the valley of the Incomáti River, which is prone to cyclical drought and flood, resulting in price fluctuation for OFSP roots (Tedesco and Brouwer 2015). In Gurúè, a higher elevation zone, participants generally emphasized drought as the main climatic constraint to production (MU = 23), though intensive flooding across Zambézia province in 2015, followed by early cessation of rainfall, led to critical concern for the food security of many communities.

Opinions on pest resistance of SP varied. Some participants reported that insect pests (sweet potato weevil [*Cylas formicarius]*), rats, and moles (likely the bushveld gerbil [*Tatera leucogaster*] or red veld mouse [*Aethomys chrysophilus*]) were major obstacles to the production of any SP, while others reported that one color suffered more than the other. Several participants believed OFSP varieties are inherently more vulnerable, while others stated that WFSP was more vulnerable due to the fact that it takes longer to mature than OFSP. Subthemes were not distinguished between pests and disease for OFSP and WFSP due to the complex nature and inconclusive data around the plant’s competitors.

Farmers in all districts emphasized the importance of seasonality and its effect on the price of both WFSP and OFSP (MU = 69). The height of SP availability varied across regions due to climate and weather patterns and crop rotations.

While SP was considered important for household consumption and/or sale by all participants, the majority reported that maize or cassava were the most important crops for their families, represented in discussions about other crops (MU = 52). Participants frequently spoke of the size and dispersal of their farm plots (MU = 90), noting that the parcels of land they cultivate are often not aggregated; rather they grow crops on multiple small plots that are located at variable distances from one another. This tradition makes it difficult for many people to estimate the size of their farms. Interviewees often stated that they had an entire hectare of SP but that they only sold a very small amount. A hectare of SP can yield, on average, 10 t of tuberous roots. Unless the entire crop was lost due to adverse environmental conditions, it is unlikely that a person selling only a few bags of SP cultivated an entire hectare, or even 0.25 ha. To better understand this seeming confusion and in order to compare responses, we often supplemented the question of farm size by asking how many bags of SP were sold in the past cycle. Farmers were able to recall the number of bags sold with relative ease; the majority had sold only a few bags of WFSP and/or OFSP. However, one farmer in Manhiça estimated that he would sell 50 sacks of 50 kg each this year. In Macate, one farmer reported selling ten 50 kg sacks of OFSP vines, while another stated that he had sold six t of WFSP roots last year.

Participants made comments on the growth patterns for both SP roots (MU = 17) and vines (MU = 12), often focusing on the time to root maturity (MU = 40). Although many participants reported that WFSP varieties develop longer vines and more abundant leaves than OFSP varieties, there was no consensus on whether this growth pattern is beneficial or detrimental. There was also a general consensus among participants that WFSP takes longer to mature than OFSP, which is confirmed by the fact that OFSP has been specifically bred to mature rapidly.

Vine preservation and retention emerged as important subthemes in this study (MU = 78 and MU = 50). The majority of participants indicated that access to planting material was a key constraint to beginning, continuing, and expanding production of OFSP. In some cases, participants have been able to preserve yellow and orange varieties for multiple years, but those who lose their planting material due to drought, flood, or lack of time/planning often report difficulty in finding vines for the next season (MU = 27).

The storage of roots was also discussed in detail (MU = 109). Several participants in Macate indicated that storage of SP is less of a priority now than it was in the past, due to the fact that SP is now typically planted and harvested multiple times annually. The increasing commercialization of SP, especially as youth and men struggle to find employment, was also cited as a reason for infrequent storage. Those who produce SP often quickly sell the majority of their crop, leaving only a small portion for consumption at home. Multiple participants noted that, although they sell SP, they also practice storage for household consumption.

Several participants reported that harvested SP can be stored longer when it is intended for home consumption rather than sale because vendors prefer fresh roots; vendors in Gurúè noted that after three days, SP is no longer good for sale. Multiple participants also mentioned that SP becomes sweeter the longer you store it. Storage methods mentioned included: 1) leaving SP in the ground and harvesting small amounts to eat or larger amounts to sell; 2) curing SP in the sunlight for one to two days before storing indoors, either in a sack or spread on a cement floor; 3) digging a hole, sterilizing the soil with fire, and storing SP inside (after curing for one to two days) covered with grass; 4) storing SP on a raised platform, covered by grass. The reported length of time for which SP can be stored varied widely, from less than a week to five months. Reports were variable on which method gave the best result; however, methods one and two were more commonly reported than methods three and four.

The use of improved agricultural techniques emerged as an important subtheme in this study (MU = 92). While some interviewees, often DVMs or project facilitators, believed that improved agricultural practices benefit any SP regardless of flesh color, other participants believed that the methods they have learned from government extension workers or NGO staff, including precise measurement between plants and planting only one vine in each hole, are specifically for OFSP and not necessary for the production of WFSP.

Participants reported on the mixing or separation of WFSP and OFSP in fields (MU = 48). Many stated that the colors should be cultivated in separate fields for a variety of reasons: 1) WFSP varieties take longer to reach maturity than OFSP; 2) WFSP varieties develop longer vines that would outcompete OFSP for space, and; 3) planting separately facilitates the harvest and sale of different flesh colors.

### Confirmation

While WFSP (MU = 34) was reported to be more common than OFSP (MU = 14), participants reported that OFSP (MU = 64) is more sought after than WFSP (MU = 1). Adults exhibited a preference for OFSP (MU = 28) over WFSP (MU = 1), including the roots and leaves, due to superior flavor; participants also reported that OFSP is preferred by children (MU = 24). Few mentioned texture or wateriness as a factor distinguishing OFSP from WFSP. This is an important finding which indicates that the 15 improved varieties of OFSP released in 2011 are compatible with typical Mozambican taste preferences, and that those who have tasted OFSP may easily be persuaded to experiment with the crop (decision) and eventually move to the implementation phase of the Innovation-Decision process.

**Fig. 6:**
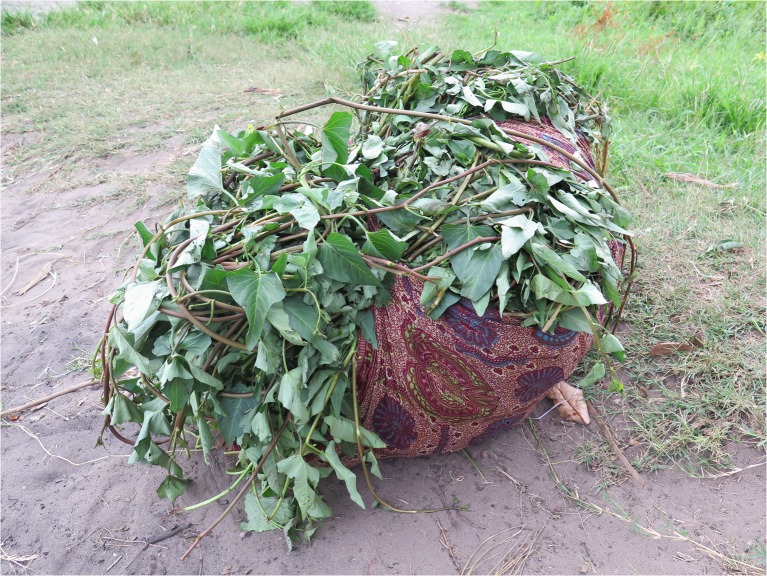
A bundle of sweet potato vines

The sale of SP roots (MU = 102) and vines (MU = 48) emerged as important subthemes in this study. In Macate, participants reported a higher rate of buying and selling vines compared to the other two districts, where vines were more often shared for free among neighbors, especially WFSP. Several DVMs reported that community members purchase vines if they have money, but that someone who does not have money will likely be given vines for free. Others reported that all vines are shared for free in order to maintain trust between neighbors, noting that anyone can lose vines and may need support in the future (Fig. 6).

In markets in all districts, SP is often sold in piles measured by sight rather than by weight. In Manhiça and Gurúè, the researcher observed vendors selling piles of SP of mixed flesh colors, varying from light yellow to dark orange. Although farmers reported planting, harvesting, and selling SP flesh colors separately, market vendors reported that the sacks they purchase from farmers often include a variety of flesh colors, especially during times of scarcity. In these cases, vendors may separate the flesh colors for resale in order to accommodate customer preferences. For many varieties, it is difficult to know the flesh color without breaking the potato or scratching the skin, which is most likely a key factor in the mixing (intentional or unintentional) of flesh colors in the same sacks; however, market vendors reported being able to distinguish the flesh color without breaking the potato.

**Fig. 7:**
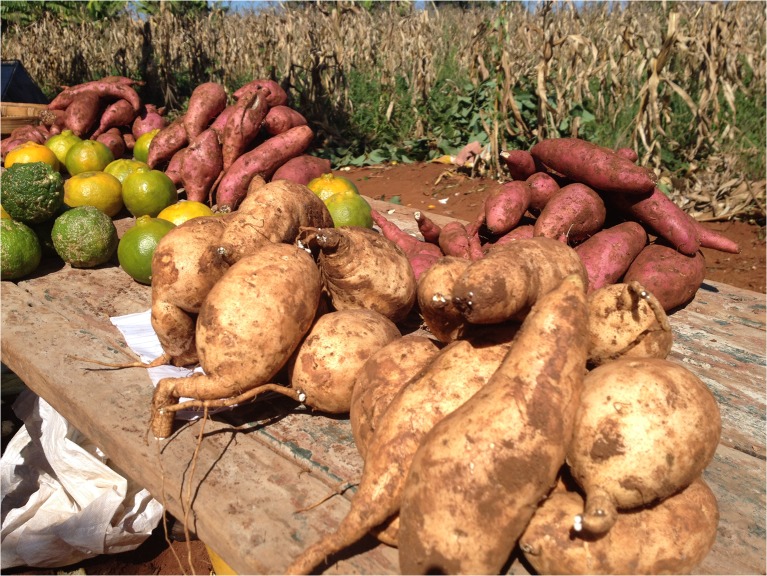
Sweet potato roots for sale

Captured across several MUs, farmers and vendors repeatedly reported that when WFSP and OFSP roots are both available at the market, OFSP is purchased faster than WFSP and at a higher price (Fig. 7). However, timing seems to be as important as flesh color, if not more so, in determining the price of SP. Several participants reported that the first SP of the year demand a higher price than the later season SP, regardless of the color.

The sharing of roots and vines (MU = 137), as well as information (MU = 21), emerged as important subthemes. Various participants described OFSP as a “novelty” and therefore something that farmers are less willing to share with one another when they first receive planting material. Their initial priority was to multiply enough vines for their own use, at which point they might share planting material with others, sometimes charging for this service. One DVM reported that producers who have access to OFSP vines may even mislead neighbors who seek planting material in order to capitalize on local markets by maintaining their status as one of the few producers of OFSP in a particular area. Hesitance to share information among some who have produced OFSP indicates that the focus may be on the individual advantage to be gained from using this crop rather than how OFSP could be distributed throughout social systems.

Participants expressed an interest in producing OFSP in the future (MU = 18), but sometimes qualified this response by noting that limited access to inputs (e.g., human or agricultural) would make expansion of the crop difficult. When asked how the project could be improved, various participants requested new varieties of OFSP, indicating a desire to experiment and continue producing OFSP in the future.

## Discussion

The results of this manuscript highlight factors that influence the adoption and retention of OSFP. These factors can be concisely determined by asking and answering questions about the motivation for producing and consuming OFSP, the differences between OFSP and WFSP, and the constraints to increased adoption of OFSP.

### Motivation to produce and consume OFSP

The participants in this study exhibited a strong understanding of the nutritional and health benefits of OFSP consumption, including the prevention of illness and positive impact on visual health. The perception that OFSP is a form of medicine, and that only individuals with outward signs of illness should consume OFSP, could potentially lead to the formation of negative attitudes (persuasion) toward OFSP among individuals who do not exhibit obvious symptoms of illness. Conversely, the belief that OFSP is medicine could lead to the formation of positive attitudes (persuasion) and increase consumption levels among those who may believe that OFSP prevents certain illnesses, increasing its relative advantage over WFSP. The health and nutrition awareness component of OFSP interventions is largely dependent on extension workers who are trained to spread messages about vitamin A and prevention of blindness.

Participants discussed market instability (MU = 24), resulting in hesitation to expand their production of SP and other crops, and voiced an interest in a processing facility that could purchase OFSP year round to ensure that their production efforts will be consistently profitable. However, this concern seems to be at odds with the insistence by other participants that OFSP is widely sought after and demands a higher price at markets. This seeming contradiction points to the complicated dynamics of OFSP supply and demand in Mozambique, and highlights the need for a comprehensive analysis of the risks and benefits, both real and perceived, among producers.

### Differences between WFSP and OFSP

The difference in culinary use of SP flesh colors noted by some participants could have a negative or positive impact on OFSP consumption levels. Several participants in Macate demonstrated confusion around the preparation of OFSP, asking “what can I do with this potato” or “how can I prepare it”, indicating that they considered it to be a distinct food from WFSP, which is typically consumed after boiling or roasting and is not used to create derivatives. The potential perception that OFSP is more difficult to incorporate into meals could result in diminished consumption of OFSP roots. However, as participants learn to incorporate OFSP into diverse recipes (e.g. enriched porridges, juice and breads), consumption levels could increase. To increase adoption, future breeding efforts to cross OFSP should build upon local knowledge about varieties with positive attributes, such as *Secai* in Macate.

The reported differences in year around availability of OFSP and WFSP is potentially attributable to the fact that OFSP varieties have been bred to mature quickly (in approximately four months), while most participants reported that WFSP takes longer to mature. The shorter maturation period for OFSP is theoretically a great advantage, and many participants spoke positively of this attribute; it could, however, result in shorter periods of availability if farmers are not willing or able to plant SP multiple times per year.

According to two subsistence farmers in Macate, WFSP is more resistant than OFSP because the vines grow longer and are therefore less susceptible to external factors (e.g. cows entering a farm) that could destroy them. However, according to one DVM, the OFSP varieties that develop shorter vines and fewer leaves are preferable because they are easier to manage. The priorities of these participants for their SP production are notably different: the subsistence farmers produce small amounts of roots mainly for consumption, and therefore appreciate vines that they perceive as less vulnerable; the DVM is concerned with efficiency in vine multiplication, and therefore appreciates vines that are easily managed. Another subsistence farmer reported that because the WFSP vines grow longer, they need more water than OFSP. She also preferred the more modest development of OFSP vines because they require smaller seedbeds than WFSP and can produce a large amount of roots in a small area. A DVM in Gurúè made a similar observation, and also noted the larger root size of OFSP compared to WFSP, which he attributed to inherent qualities rather than planting technique.

Several participants reported that OFSP could be preserved on the farm for a shorter period of time than WFSP due to high susceptibility to pests and poor resistance to sun. Others believe that WFSP lasts longer post-harvest than OFSP. The formation of such beliefs during the implementation phase could lead participants to reject OFSP in the confirmation phase if the perceived advantages of taste, health, and profitability do not outweigh the agronomic challenges associated with OFSP.

Multiple participants reported that OFSP requires the use of improved agricultural techniques which are not required for the production of WFSP. Further, the need to remove all weeds prior to planting OFSP was cited by multiple participants and seems to be a deterrent for some producers. This perceived need for a “clean plot” and careful measurement can result in the belief that OFSP is more work than WFSP, thereby creating a barrier to adoption. Conversely, some participants reported that OFSP is less work because the vines are smaller and the roots mature faster, necessitating less frequent weeding. Multiple participants also mentioned that OFSP can be harvested by hand while WFSP needs to be harvested with a hoe, as its roots grow deeper into the soil. Future research should examine the potential differences in root systems between WFSP and OFSP as this could have implications for the plant’s ability to access water stored in the soil, as well as the difficulty or ease of harvest.

### Constraints to increased adoption of OFSP

Although DVMs and other participants acknowledged the positive health properties of OFSP and indicated a sense of pride associated with its production, they also reported low levels of motivation among some community members to produce OFSP, and especially to preserve vines. Participants frequently mentioned a need for incentives such as branded t-shirts and *capulanas* (colorful, patterned fabric), as well as vine distributions, nutrition education, agricultural inputs and market support in order to produce OFSP. DVMs reported that community members need encouragement to produce OFSP again in the future if their crop is unsuccessful in one year; some DVMs believe that without their own continued efforts to preserve and distribute vines, it would be very unlikely that OFSP would persist in their communities. This concern is reinforced by the fact that multiple participants noted that WFSP is “easier” to produce due to a perceived greater need for field preparation and weed maintenance for OFSP.

Evidence of a lack of adoption of OFSP is also seen in the contrasting colloquial terminology for WFSP versus OFSP and the lack of ability of farmers to recall OFSP nomenclature. However, a growing trend to rename OFSP using local nomenclature is a form of reinvention that may potentially lead to increased adoption and sustainability (Rogers 2003).

A portion of participants reported that both OFSP and WFSP vines must be transferred to the moist lowlands during the dry season to preserve planting material for the following season as drought affects all flesh colors equally; others reported that vines may at times be preserved on-farm, depending on the extremity of temperature and dryness. However, multiple participants reported that OFSP vines must be transplanted to the humid lowlands during the dry season, while WFSP can survive in the higher elevation fields and does not require transfer to moist soils. In fact, several respondents reported that white vines they have ignored, or even actively tried to remove, have still independently germinated year after year. Participants in Gurúè also emphasized that flooding followed by insufficient rain resulted in very low yields of OFSP roots, while WFSP roots were still available, adding strength to claims that WFSP varieties are more drought resistant. These are key findings that highlight an important challenge associated with achieving a critical mass of OFSP in Mozambique. Farmers who have recently begun to produce OFSP, who have not yet confirmed it as a useful technology, may ultimately reject the crop if it is perceived as less tolerant to unpredictable climate and weather patterns than WFSP.

A key perceived benefit to preserving vines articulated by one DVM in Gurúè is that the practice enables farmers to produce and sell SP before other farmers who did not preserve vines and therefore must purchase planting material or depend on the support of others who may only consent to share vines once their own fields have been planted. Farmers who reported high levels of vine retention stated that those who lose their vines and must search for new planting material each season engage in this behavior for several reasons: 1) they do not have access to humid lowland zones, and therefore cannot transfer vines for preservation during the dry season; 2) they do not make the effort to maintain planting material because they know they can get it from a neighbor or organization the following season; 3) they are careless in their harvesting, perhaps pushing sand over the vines where they collected the roots in hopes that some might survive for the coming season instead of carefully replanting the vines. This practice was reported to result in a higher survival rate for WFSP than for OFSP.

Leaf consumption was discussed frequently in interviews and focus groups (MU = 49); one interviewee in Gurúè reported that some families consume the majority of the SP leaves when other food is scarce, resulting in a low survival rate for vines. Still others may be overly generous in the sharing of vines or leaves with friends and neighbors, leaving them with little planting material for their own production.

Two DVMs in Gurúè mentioned difficulties enforcing the CIP Viable Sweetpotato Technologies for Africa (VISTA) project policies on monetary contributions for vines. One reported that families complained that vines cost two meticais (USD $0.03) for six kg due to the knowledge that families benefiting from former dissemination efforts received eight kg for free. Another reported that he could not collect the two meticais contribution as community members are accustomed to receiving planting material for free; he therefore had to give away vines to community members in order to accomplish his dissemination goals. This resistance to monetary contributions, even among those who have received information about VAD and the nutritional value of OFSP, may suggest that the perceived advantage of OFSP is not as strong as the conviction that vines should be shared for free, as is customary in many communities.

The study results show that both men and women in all three districts engage in the cultivation, preservation, and sale of OFSP. Due to the variability of responses in each district to questions about gender roles, further research should be conducted to understand geographically specific norms as a factor affecting the adoption or rejection of OFSP in Mozambique. Gender norms have important implications in the implementation phase of any new innovation; a possible strategy for future OFSP interventions could be to increase focus on the role of women as vendors of SP, especially in Macate where the crop is perceived as highly important for business.

## Study limitations

Most interviews and focus groups were conducted in remote areas where some participants did not speak Portuguese, necessitating a reliance on translators, which has the potential to influence research findings (Temple 1997). Further, as the first author often traveled by motorcycle, bicycle, or on foot to conduct research, she was unable to work with a single translator to assist with local language, which varied among districts. The first author would not have been able to meet participants in remote areas without the assistance of extension workers and NGO facilitators, and participant comfort levels as well as honest reporting were potentially increased due to familiarity with these individuals as translators. Conversely, it is possible that the presence of these individuals led to misreporting in some cases. The researcher identified some participants independently, but in other cases the participants were identified by extension workers, project facilitators, or other participants. This method of sampling substantially broadened the geographic area included in the research, but also could have created a bias in the characteristics of participants. Risks of the sampling strategy include bias during informant selection, as the researcher judged the informants’ reliability, and the potential for informants to misreport due to favoring the socially desirable response. Self-reporting by participants could also have led to misreporting in some cases, especially in attempts to estimate plot sizes and recall the amount of SP sold in the past season. These risks were controlled by taking several entry points to initiate ‘snowball sampling’ and crosschecking the responses through triangulation with other participants’ responses and the researcher’s detailed field log (Creswell 2014; Tongco 2007). Finally, meaning units were analyzed at the fragment level instead of at the participant level. There are advantages and disadvantages to each method. At the fragment level, the summation of the qualitative work is analyzed to reach a common understanding of all participants; at the participant level, the differences in individual participant responses are analyzed.

The original goal of the research was to interview 20 participants in each research site, and this goal was surpassed in each district. However, due to project logistics, half of the participant sample lived in Macate, which has the potential to bias study results. Researchers analyzed data by district to understand potential differences and noted several in the study results. Few in-person follow-up interviews were conducted due to time and transportation constraints; however, many participants were accessible by phone and the researcher made multiple calls to ask follow-up questions.

## Conclusion

Results from this research indicate that a wide variety of factors influence the adoption and retention of OFSP across the Diffusion of Innovations model. These include: perceptions of OFSP as different from WFSP in terms of nutrition, organoleptic qualities, and culinary properties; taste preferences for both roots and leaves; access to planting material; perceived difference in agronomic traits, including pest and drought resistance, time to root maturity, vine development, and post-harvest storage capacity; perceptions that OFSP is more work due to improved agricultural practices; dependence on NGOs or neighbors for planting material; perceptions of OFSP as the more economically competitive SP; inability to increase production due to lack of access to capital for inputs and labor; unstable markets, fluctuating prices, and relationships with traders, and; varying levels of sharing of information and planting material across farmer networks.

Access to planting material is a crucial factor as study participants demonstrated willingness to produce, consume, and sell OFSP. Dependence on government and NGOs for vine distributions may be diminished if the number of individuals multiplying vines, independently or as contracted DVMs, is increased. However, incentives for vine multipliers remain a challenge as it is clear that many Mozambicans are accustomed to sharing WFSP vines freely and are thus hesitant to pay for planting material.

Understanding that some individuals prioritize vine preservation while others tend to seek assistance from neighbors or organizations is an important aspect of planning future interventions to further the diffusion of OFSP in Mozambique. As OFSP becomes more available, perceptions of the crop as a “novelty” may wane, which could have a positive effect on sharing of information and planting material. Agronomic traits that distinguish WFSP from OFSP, including drought tolerance and time to root maturity, have a key influence on the utility of OFSP, as do perceptions that OFSP requires improved agricultural practices that are not necessary for the production of WFSP.

Based on these findings, we suggest the following measures to ensure continued growth in adoption levels of OFSP:

1. Future programs should consider enabling DVMs to provide vines at no charge in communities where residents are accustomed to sharing vines freely and are hesitant to pay for planting material. Although it would require program inputs, this approach could potentially result in an increase in year-round availability of OFSP vines, reduce any existing perceptions that OFSP is a novelty crop that should not be shared openly, and eventually lead to OFSP reaching a critical mass. Vouchers may be distributed to community members to facilitate the acquisition of vines, and may be phased out over time if assessment shows that communities are willing and able to purchase vines. In addition to vine production, DVMs should be encouraged to allow some plants to grow to full maturity for consumption and/or sale of the roots.
2. Training should emphasize the similarities in agronomic practices required for producing and preserving OFSP and WFSP (e.g., removing weeds or planting in rows) in order to dispel the perception that OFSP is more labor intensive. Training should also reiterate the difference in time to root maturity between WFSP and OFSP, as OFSP has been bred to mature more quickly. This knowledge will enable farmers to stagger plantings and prepare for multiple annual cycles of OFSP, whereas WFSP is traditionally produced only once per year.
3. Based on multiple accounts from farmers that WFSP is more drought tolerant, plant breeders should continue to focus on creating more drought tolerant varieties of OFSP, as well as the wider distribution of relatively new OFSP varieties which may be equally drought resistant to WFSP but have not yet reached all the farmers included in this study. Future breeding efforts may also consider reports from this research that OFSP cannot be preserved as long as WFSP post-harvest. Continued efforts should also be made to discover and incorporate popular local varieties of white and yellow SP, such as *Secai*, into future breeding trials.
4. The nutritional and agronomic benefits of growing OFSP are well known by many farmers; in particular, many reported that OFSP is good for health, good for the eyes, and that OFSP roots mature faster than WFSP. These favorable traits should continue to be emphasized when introducing new OFSP varieties, and especially when training DVMs. Further awareness raising around OFSP as a food that can help prevent vitamin A deficiency and promote healthy eyes and vision should be conducted in schools and community centers.
5. Many respondents reflected positively on past experiences with OFSP cooking demonstrations, and also reported a preference for the flavor of OFSP roots and leaves. Program implementers should continue to offer such demonstrations, focusing on the versatility of SP (i.e. not only a breakfast food) and including savory recipes and derivatives (e.g. juice and breads), as well as preparations of SP leaves involving other nutritious, locally available ingredients.
6. Farmers and vendors of SP should be encouraged to separate OFSP from WFSP as there is evidence from a recent report (Tedesco and Brouwer 2015), as well as from participants and market observations in this study, that OFSP commands a higher price at many markets. Consumer surveys should be conducted and the findings shared in order to better understand awareness of the health benefits of OFSP, consumption patterns and market opportunities. Program implementers may also consider working to improve relationships between farmers and traders, which may be facilitated by ensuring that farmers have access to up-to-date information on market price and availability.

One participant’s quote summarizes the importance of OFSP as a mechanism for the prevention of Vitamin A deficiency (VAD) in Mozambique, indicating that the crop will continue to be produced in the future: [translator] “Yes she is thanking … the orange pulp. She says a long time ago, the people didn’t have strength, the children, you are just seeing your child getting thin. But now after orange pulp appeared, the development [of children], it’s different. It’s different because, a person when they see that ‘well, these days I don’t have strength’ you have that way to prepare the orange pulp, eat it, they are well. They have small children, you, always in each house, prepare, and give it to the children, and the child grows very healthy. Yes yes. Then for this that until today, the orange pulp no, the people don’t leave it, because it helps us well in health.”

Further research should be conducted to understand the pest tolerance and vulnerability for newer varieties of OFSP in regionally specific contexts, as participant responses on this topic were variable. Better understanding of farmer preference for vine and leaf development could guide future breeding efforts, as some farmers prefer varieties with more abundant leaves and vines while others prefer varieties with more modest vine growth. A cost-benefit analysis comparing OFSP to beta-carotene-rich indigenous crops, considering the cost of labor, transport, and other inputs, would also be useful to increase understanding of farmer incentives to produce and sell OFSP. Finally, an in-depth understanding of how gender dynamics relate to SP production and commercialization is key to improving markets and supporting female vendors, who have been shown to dominate the retail of SP in both rural and urban markets (Tedesco and Brouwer 2015).

The cultivation of vitamin A-rich OFSP is an important tool for preventing VAD and increasing food security in resource-poor communities in various country contexts. Organizations like HarvestPlus and the International Potato Center are working diligently to promote this vitamin-A-rich staple food in multiple countries across Africa and Latin America and have made great strides in improving the availability and consumption of OFSP. A recent study that examined perceived benefits of producing and consuming OFSP among farmers in Malawi found that multiple perceived health and economic benefits, including increased energy for work and the cognitive development of children, were key determinants in the adoption of OFSP (Mudege et al. 2017). Another study in Kenya found that using health services as a platform to promote OFSP to pregnant and lactating women is a feasible strategy for improving nutrition knowledge, vitamin A intakes, and vitamin A status (Girard et al. 2017).

The majority of the 95 participants in our study exhibited highly positive attitudes towards OFSP based on their experiences as producers, consumers, and vendors. Addressing the opportunities and challenges described in this research could lead to an increase in the adoption and retention of OFSP varieties in Mozambique and other geographic locations in sub-Saharan Africa where the crop has been introduced.

In their contribution to The Lancet series on Maternal and Child Nutrition, Ruel and Aldermann (2013) noted the feasibility and effectiveness of OFSP for improving maternal and child intake of vitamin A and child vitamin A status; the article stated that biofortification of OFSP provides the most conclusive evidence to-date of the effect of an agricultural program on nutrition outcomes. Findings from the current research outline key insights into the uptake of OFSP as well as clear strategies that can also be applied to the introduction of other biofortified crops. The lessons learned from over 15 years of work to breed, disseminate, and multiply OFSP in Mozambique and other sub-Saharan African countries provide an excellent framework for other nutrition-sensitive agriculture approaches designed to increase agricultural productivity, improve dietary diversity, and alleviate micronutrient malnutrition, thereby improving the nutrition and food security of communities.

## Footnotes

1 In the context of OFSP, critical mass could be measured by tangible aspects such as decreased need for technical assistance, decreased reliance on organizations or neighbors for planting material, common food product identified in dietary recalls, decrease in negative health conditions related to vitamin A deficiency, and increased availability in markets.

## References

[CR1] Aguayo V, Kahn S, Ismael C, Meershoek S (2005). Vitamin a deficiency and child mortality in Mozambique. Public Health Nutrition.

[CR2] Bai C, Twyman R, Farré G, Sanahuja G, Christou P, Capell T (2011). A golden era—pro-vitamin A enhancement in diverse crops. In Vitro Cellular & Developmental Biology. Plant.

[CR3] Brito L, Brouwer R, Falcão M (2012). Sweetpotato—Biotechnology in different guises on a broad range of scales. Technological Forecasting and Social Change.

[CR4] Creswell JW (2014). Research design.

[CR5] de Brauw A, Eozenou P, Gilligan DO, Hotz C, Kumar N, Loechl C, McNiven S, Meenakshi JV, Moursi M (2010). The impact of the HarvestPlus reaching end users orange-fleshed sweet potato project in Mozambique and Uganda.

[CR6] de Carvalho IST, Tivana LD, Granfeldt Y, Dejmek P (2014). Improved energy and sensory properties of instant porridge made from a roasted mixture of grated orange-fleshed sweet potatoes and flour made from shredded sun dried cassava. Food and Nutrition Sciences.

[CR7] FAOSTAT. (2015). Production Statistics for Sweet Potato. http://faostat.fao.org/site/339/default.aspx. Accessed November 7, 2015.

[CR8] Girard AW, Grant F, Watkinson M, Okuku HS, Wanjala R, Cole D, Levin C, Low J (2017). Promotion of orange-fleshed sweet potato increased vitamin a intakes and reduced the odds of low retinol-binding protein among postpartum Kenyan women. The Journal of Nutrition.

[CR9] HarvestPlus (2012). Disseminating Orange-fleshed sweet potato: Mozambique country report.

[CR10] Herforth A, Jones A, Pinstrup-Andersen P (2012). Prioritizing nutrition in agriculture and rural development: Guiding principles for operational investments. Health, nutrition and population (HNP) discussion paper.

[CR11] Hotz C, Loechl C, de Brauw A, Eozenou P, Gilligan D, Moursi M, Munhaua B, van Jaarsveld P, Carriquiry A, Meenakshi JV (2012). A large-scale intervention to introduce orange sweet potato in rural Mozambique increases vitamin a intakes among children and women. The British Journal of Nutrition.

[CR12] Jenkins M, Byker Shanks C, Houghtaling B (2015). Orange-fleshed sweet potato: Successes and remaining challenges of the introduction of a nutritionally superior staple crop in Mozambique. Food and Nutrition Bulletin.

[CR13] Jones KM, de Brauw A (2015). Using agriculture to improve child health: Promoting orange sweet potatoes reduces diarrhea. World Development.

[CR14] Kapinga R, Andrade M, Lemaga B, Gani A, Crissman C, Mwanga R (2005). Role of orange-fleshed sweetpotato in disaster mitigation: Experiences from east and southern Africa. African Crop Science Conference Proceedings.

[CR15] Krippendorff K (2004). Content analysis: An introduction to its methodology.

[CR16] Labarta, R. (2009). Are small sub-Sahara African farmers willing to pay for vegetative propagated orange fleshed sweetpotato planting material? Evidence from Central Mozambique. Proceedings of the Agricultural & Applied Economics Association AAEA & ACCI joint annual meetings; July 2009; Milwaukee, WI.

[CR17] Low, J. (2013). Biofortified crops with a visible trait: The example of orange-fleshed sweetpotato in sub-Saharan Africa. In V. Preedy, R. Srirajaskanthan, V. Patel (Eds.), *Handbook of food fortification and health: From concepts to public health applications* (pp.371–384). Vol 1. New York: Springer Science+Business Media.

[CR18] Low J, Uaiene R, Andrade MI, Howard J (2000). Orange-flesh sweet potato: Promising partnerships for assuring the integration of nutritional concerns into agricultural research and extension in Mozambique.

[CR19] Low, J., Arimond, M., Osman, N., Osei, A.K., Zano, F., Cunguara, B., et al. (2005). Toward sustainable nutrition improvement in Mozambique: Addressing macro- and micro-nutrient malnutrition through new cultivars and new behaviors: Key findings. East Lansing: Michigan State University.

[CR20] Low J, Arimond M, Osman N, Cunguara B, Zano F, Tschirley D (2007). A food-based approach introducing orange-fleshed sweet potatoes increased vitamin a intake and serum retinol concentrations in young children in rural Mozambique. The Journal of Nutrition.

[CR21] Low J, Arimond M, Osman N, Cunguara B, Zano F, Tschirley D (2007). Ensuring the supply of and creating demand for a biofortified crop with a visible trait: Lessons learned from the introduction of orange-fleshed sweet potato in drought-prone areas of Mozambique. Food and Nutrition Bulletin.

[CR22] Low J, Arimond M, Labarta R, Andrade M, Namanda S, Fanzo J, Hunter D, Borelli T, Mattei F (2013). The introduction of orange-fleshed sweetpotato (OFSP) in Mozambican diets: A marginal change to make a major difference. Diversifying food and diets: Using agricultural biodiversity to improve nutrition and health.

[CR23] MAE (2005). Perfil do distrito de Manhiça.

[CR24] MAE (2005). Perfil do distrito de Gondola.

[CR25] MAE (2005). Perfil do distrito de Gúruè.

[CR26] Marshall MN (1996). Sampling for qualitative research. Family Practice.

[CR27] Minde, I., & Jumbe, C. (1997). Situation analysis and outlook of cassava and sweet potato in SADC countries. Technical report no. 5. Study Report Prepared for SADC/IITA/SARRNET, SARRNET.

[CR28] Morgan DL (1997). Focus groups as qualitative research.

[CR29] Mudege NN, Mayanja S, Muzhingi T (2017). Women and men farmer perceptions of economic and health benefits of orange fleshed sweet potato (OFSP) in Phalombe and Chikwawa districts in Malawi. Food Security.

[CR30] Mwanga ROM, Odongo B, Niringiye C, Alajo A, Kigozi B, Makumbi R (2009). ‘NASPOT 7’, ‘NASPOT 8’, ‘NASPOT 9 O’, ‘NASPOT 10 O’, and ‘Dimbuka-Bukulula’ sweetpotato. HortScience.

[CR31] Naico A, Lusk J (2010). The value of a nutritionally enhanced staple crop: Results from a choice experiment conducted with orange-fleshed sweet potatoes in Mozambique. Journal of African Economies.

[CR32] O’Brien PJ (1972). The sweet potato: Its origin and dispersal. American Anthropologist.

[CR33] Rogers E (2003). Diffusion of innovations.

[CR34] Ross JS (1996). Derivation of the relative risk of child mortality due to vitamin a deficiency. PROFILES working notes series no. 2.

[CR35] Ruel MT, Aldermann H (2013). Nutrition-sensitive interventions and programmes: How can they help to accelerate progress in improving maternal and child nutrition?. The Lancet.

[CR36] Sommer A, West K (1996). Vitamin a deficiency: Health, survival and vision.

[CR37] Tedesco I, Brouwer R (2015). Sweetpotato value chain and the potential role for commercial fresh root storage in selected areas of Mozambique.

[CR38] Temple B (1997). Watch your tongue: Issues in translation and cross-cultural research. Sociology.

[CR39] Temple B, Young A (2004). Qualitative research and translation dilemmas. Qualitative Research.

[CR40] Tongco DC (2007). Purposive sampling as a tool for informant selection. Ethnobotany Research and Applications.

[CR41] Walker T, Pitoro R, Tomo A, Sitoe I, Salência C, Mahanzule R (2006). Estabelecimento de prioridades para a investigação agrária no sector público em Moçambique baseado nos dados do trabalho de inquérito agrícola (TIA). Relatório de pesquisa no. 3P.

[CR42] Wertz FJ (1983). From everyday to psychological description: Analyzing the moments of a qualitative data analysis. Journal of Phenomenological Psychology.

[CR43] WHO. (2009). Global prevalence of vitamin A deficiency in populations at risk 1995–2005. WHO global database on vitamin a deficiency. Geneva: World Health Organization.

[CR44] Woolfe J (1992). Sweet potato: An untapped food resource.

